# Hyaluronic Acid: Exploring Its Versatile Applications in Dentistry

**DOI:** 10.7759/cureus.46349

**Published:** 2023-10-02

**Authors:** Abhinn Miglani, Rozina Vishnani, Amit Reche, Janhavi Buldeo, Bhinika Wadher

**Affiliations:** 1 Department of Periodontology and Oral Implantology, Datta Meghe Institute of Higher Education and Research, Wardha, IND; 2 Department of Oral and Maxillofacial Surgery, Datta Meghe Institute of Medical Sciences Sharad Pawar Dental College, Wardha, IND; 3 Department of Public Health Dentistry, Datta Meghe Institute of Higher Education and Research, Wardha, IND; 4 Department of Oral and Maxillofacial Surgery, Datta Meghe Institute of Higher Education and Research, Wardha, IND

**Keywords:** dentistry, aesthetics, augmentation, healing, regeneration, polysaccharide, hyaluronic acid

## Abstract

Hyaluronic acid (HA) a polysaccharide present in many areas of the body like the synovium of synovial joints, and connective tissues which have high regenerative and biocompatible properties has been an area of interest since recent times in dentistry. Several research papers and review articles were studied in the Pubmed database to formulate this review article. The main aim of this article is to demonstrate various applications of HA in different branches of dentistry. The PubMed database was searched for keywords “Hyaluronic acid AND periodontics,” “Hyaluronic acid AND oral and maxillofacial surgery,” “Hyaluronic acid AND oral medicine,” “Hyaluronic acid AND orthodontics,” “Hyaluronic acid AND endodontics,” and “Hyaluronic acid AND aesthetic dentistry” which resulted in six, 296, 83, 86, 40, and 49 articles, respectively. The most relevant and informative articles were selected and studied for this review article. This review article will also help people to gain knowledge about the future aspects of the use of HA in dentistry and also motivate clinicians and new-generation dentists to inculcate the HA’s use in their respective practice in dentistry.

## Introduction and background

The human body contains hyaluronic acid (HA), a glycosaminoglycan that occurs naturally in various tissues like the skin, joints, eyes, etc. Its biocompatibility and regenerative properties have made it a topic of great attention and interest in the field of dentistry. Among its physiological and structural functions are its extracellular and cellular interconnections, relationships with growth factors and osmotic pressure control, tissue lubrication, and osmotic pressure regulation henceforth contributing to the structural and homeostatic integrity of the tissue. Many investigations by Kingsley M, Dahiya P, and many others into the physiological function of HA in humans and its chemical and physicochemical characteristics have shown its suitability as a biomaterial for use in pharmaceutical, medicinal, and aesthetic procedures [1,2]. Its biocompatibility, regenerative properties, and antimicrobial properties make it an attractive material for use in various dental procedures. The PubMed database was searched for keywords “Hyaluronic acid AND periodontics”, “Hyaluronic acid AND oral and maxillofacial surgery”, “Hyaluronic acid AND oral medicine”, “Hyaluronic acid AND orthodontics”, “Hyaluronic acid AND endodontics”, “Hyaluronic acid AND aesthetic dentistry”, which resulted in six, 296, 83, 86, 40, and 49 articles, respectively. The most relevant and informative articles were selected and studied for this review article. The purpose of this review article is to investigate and address the diverse physiochemical, biochemical and pharmaco-therapeutic uses/applications of HA, particularly in connection to several areas of dentistry.

## Review

Search methodology

**Figure 1:**
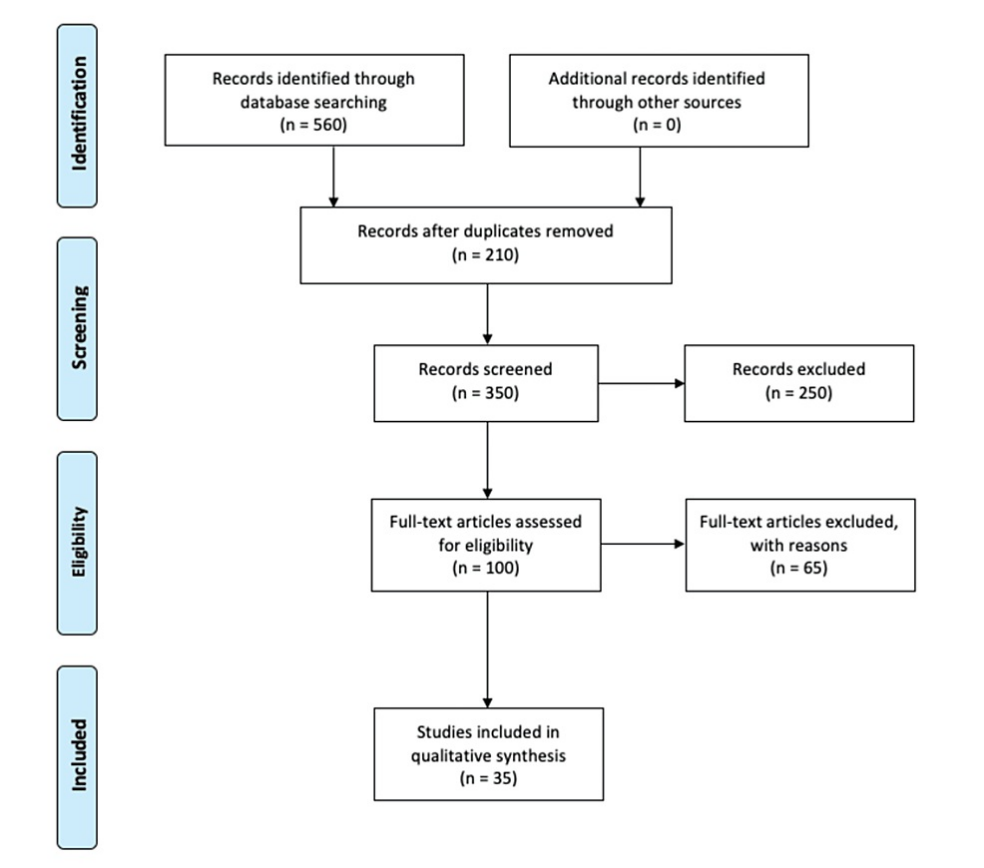
PRISMA flow diagram for literature search

We conducted a review through PubMed using keywords such as “hyaluronic acid AND periodontics,” “hyaluronic acid AND oral and maxillofacial surgery,” “hyaluronic acid AND oral medicine,” “hyaluronic acid AND orthodontics,” “hyaluronic acid AND endodontics,” “hyaluronic acid AND aesthetic dentistry.” We additionally searched for key references from bibliographies of the relevant studies. The search was updated in June 2023. The studies available in English language only and available as full texts were retrieved against the inclusion and exclusion criteria. We excluded studies published in other languages because of resource limitations and unavailability of full-text articles. Figure 1 describes the PRISMA flow diagram for the article search.

History

HA is a high molecular weight bio-polysaccharide found in the vitreous body of cow's eyes discovered by Columbia University's Karl Meyer along with assistant John Palmer in 1934 and classified as a mucopolysaccharide. It is a naturally occurring polymer found in connective tissues all over the body, including the skin, joints, and eyes. This discovery was made in the years that followed after several studies on the characteristics of HA and its function in the body. HA was also revealed to serve a significant function in wound healing. It aids in the regulation of inflammation, hence encouraging tissue healing. Also, it is an essential constituent of the extracellular matrix, which is a network of proteins and other substances that gives structural support to cells and tissues [3]. Nowadays, HA is widely utilized in medicine, dentistry and cosmetics. It is used in medicine as a joint lubricant and a ler for soft tissue augmentation. It is used in cosmetics as a moisturizer, and anti-aging agent and has also been added to hair care products as it increases skin moisture and diminishes the appearance of fine lines and wrinkles.

Chemistry

HA (also known as hyaluronan) is a polymer consisting of repeating disaccharide units that is a linear polysaccharide. HA's molecular structure is made up of alternating units of D-glucuronic acid and N-acetyl-D-glucosamine connected by -1,3 and -1,4 glycosidic linkages. The chemical formula of HA is C_14_H_21_NO_11_, and its molecular weight varies based on the length of the polymer chain. Some of its chemical properties are that it is first of all hydrophilic, which means it binds to water molecules strongly. Moreover, it can also be referred to as a polymer that has a very high molecular weight which ranges from one to 10 million Daltons. Because of the carboxylic acid groups in the glucuronic acid units, it possesses a significant negative charge. Due to its unique capacity to attach to a wide range of molecules, including proteins, lipids, and carbohydrates, it is an essential component in many biological processes. It has good biocompatibility and biodegradability, making it suitable for use in a variety of medicinal applications. It forms a gel-like structure when comes in contact with water, giving it viscoelastic qualities. Also, it can be chemically changed to improve its qualities such as improving its stability, extending its stay in the body, or enhancing cell adhesion.

Hyaluronidase

Hyaluronidase is an enzyme that can degrade HA, a chemical present in the extracellular matrix of connective tissues. In dentistry, hyaluronidase is occasionally used to assist in dissolving and dispersing injectable dermal fillers, especially if the filler was accidentally injected into a blood artery or if the patient is suffering difficulties with the filler. In addition to its usage with dermal fillers, hyaluronidase may be employed in some dental treatments [4]. It may, for example, be used as an adjuvant to local anesthesia to improve the diffusion and efficiency of the anesthetic solution. Hyaluronidase has also been examined for its possible application in the treatment of illnesses such as periodontitis and temporomandibular joint (TMJ) problems.

Chemical structure of hyaluronidase

Hyaluronidase is a protein with a chain of amino acids folded into a unique three-dimensional structure that allows it to catalyze the breakdown of HA. The precise amino acid sequence and three-dimensional structure of hyaluronidase differ between species and organism types. There are multiple types of hyaluronidases in humans, each with a slightly distinct amino acid sequence and structure [5]. All kinds of hyaluronidase, however, exhibit some structural similarities, such as an active site that binds to HA and catalyses its breakdown.

Properties of HA

HA is hygroscopic, i.e., it can absorb and retain water molecules which allows HA to act as a lubricant and shock absorber in the body, protecting tissues from mechanical stress. It is biocompatible, i.e., it is well tolerated by living tissues and doesn’t cause any adverse reactions or immune responses. HA is non-immunogenic, which means it does not stimulate an immune response or cause inflammation when injected into the body [6]. HA has a high viscosity, which makes it an effective lubricant and shock absorber in joints and other tissues. It is biodegradable, which means it can be broken down and eliminated from the body over time. It has also been shown to possess anti-inflammatory effects, which may contribute to its ability to reduce pain and swelling in arthritic joints. It also promotes tissue regeneration and repair, making it useful in wound healing and tissue engineering applications [7]. HA is widely used in cosmetics and skin care products due to its ability to attract and retain water molecules, providing a moisturizing effect on the skin.

Functions of HA

Hydration

Since HA can store 1,000 times its weight in water, it is a good hydrator for the skin and other tissues.

Lubrication

HA helps in joint lubrication thereby allowing smooth movement friction between them.

Wound Healing

HA leads to cell migration and proliferation thereby causing wound healing.

Anti-inflammatory

It helps reduce swelling and pain in the body because has anti-inflammatory properties.

Eye Health

HA is present in the eye's vitreous humour and helps to maintain its shape and integrity.

Bone Health

HA is involved in the formation and maintenance of bone tissue and has been shown to help prevent bone loss in some studies. Overall, HA plays a crucial role in maintaining the health and functioning of various tissues and organs in the body.

Common methods of application of HA in dentistry

It promotes healing and minimizes inflammation, HA can be administered topically to the oral mucosa, gums, or other soft tissues. HA can be injected directly into soft tissues to enhance them, such as in the treatment of gingival recession or to improve a patient's smile's attractiveness. It can also be injected around dental implants to boost osseointegration and implant success rates. To stimulate tissue regeneration and minimize inflammation, HA can be made into a gel or film and administered to the afflicted region. To assist in reducing periodontal infections and improving healing, HA can be added to a mouthwash or rinse [8].

HA in Oral and Maxillofacial Surgery

It can be injected into soft tissue to enhance volume and improve the area's appearance. HA has been found to improve wound healing by increasing tissue regeneration, decreasing inflammation, and increasing angiogenesis. It can help with recovery following oral procedures like tooth extractions or implant placement surgeries. In cases where bone regeneration is desired, it has been found to increase bone density and induce osteogenesis in the maxillofacial area. It can be used to improve the results of surgeries which use bone grafting [9]. Various temporomandibular disorders can be treated by injecting HA into the joint space. It assists in decreasing inflammation, enhances joint lubrication, and stimulates tissue regeneration. There is a lot of research in the literature elaborating on the use of hyaluronidase in TMJ problems. The symptoms of joint disturbances are of different types. Many authors have classified these disorders under varied classifications. Costen et al. [10] described it as a series of symptoms that involve otalgia, headache, nausea, dizziness, and deafness, and finally referred to all these symptoms leading to the loss of vertical dimension. Mullen et al. identified a situation in which the disc was ripped either anteriorly or posteriorly and was accompanied by a cracking sound [11]. Shapiro et al. described a similar condition which was characterised by varying types of sounds like cracking, grating or clicking sounds and referred to this as “partial dislocation” [12]. Gerry et al. described joint contusion, joint strain and disc derangement in their paper where joint contusion was characterized by discomfort, oedema, restriction of mobility, and mandibular deviation to the injured side [13]. Bellinger et al. also described similar conditions and termed them “arthroses” [14]. After many research and studies, many authors also described conditions in the joint region as “tenderness to palpation,” “subluxation,” and “vague pain” in the maxilla and mandible and a “buzzing” or “fullness” in the ear. Hyaluronidase is an enzyme that hydrolyses the HA that is present in joint spaces and the synovial fluid and is proven to increase the viscosity of the synovial fluid. Hyaluronidase acts by increasing the fluid absorption and synovial permeability henceforth letting the dispersion of the traumatic exudates and transudates. Several of these joint problems were thought to be characterized by oedema, increased tension, and tissue disintegration inside the joint area, and it was thought that these instances may be treated with hyaluronidase injections. Forty-two instances of TMJ problems, including restricted mobility, jaw deviation, clicking and locking, moderate subluxation, and discomfort, were treated with hyaluronidase injections of 150 TRU. There was a significant improvement in all instances treated in this series, and there has been no return of symptoms. HA is found to be of great use when it comes to its benefit in using it after third molar surgery. Yilmaz et al. researched to determine the effectiveness of administering local HA after surgically removing an impacted third molar and measuring discomfort, edema, and difficulties opening the mouth if any [15]. Twenty-five healthy individuals between the ages of 18 and 29 had asymptomatic bilaterally impacted lower third molars. The procedures were carried out under local anesthesia. The research group received 0.8% HA in the post-extraction sockets of the right third molars, while the control group received no treatment in the extraction sockets of the left third molars. During the first, third, and seventh postoperative days, discomfort, trismus, and oedema were assessed. There was no difference between groups in terms of face edema or maximal mouth opening. Yet, according to the visual analogue scale, the quantity of discomfort was considerably reduced in the HA groups (p = 0.001). According to the findings of this study, HA can induce an analgesic effect in post-extraction sockets following surgical removal of impacted teeth, and therefore it has the therapeutic advantage of reducing the need for nonsteroidal anti-inflammatory medicines after dentoalveolar surgery. Koray et al. carried out research to compare the efficacies of two oral sprays in decreasing oedema, discomfort, and trismus following the extraction of impacted mandibular third molars [16]. In terms of decreasing edema and trismus, the HA spray was more effective than the benzydamine hydrochloride spray. Although no indication of pain reduction was found, HA appears to be helpful in the treatment of oedema and trismus in the early postoperative period following impacted third molar surgery.

HA in Periodontal Therapy

HA's significance in periodontal treatment has been thoroughly researched. It possesses anti-inflammatory, anti-bacterial, anti-edematous and tissue regeneration abilities. According to studies, using HA in periodontal treatment can result in reduced pocket depth, better attachment levels, and less bleeding on probing. Additionally, HA contains antibacterial characteristics that can aid in the treatment of periodontal diseases. It can be injected into the gum tissue to encourage tissue regeneration and minimise inflammation, assisting in the restoration of the gum tissue's natural position. It can also be used in pocket reduction therapy- Periodontal pockets, which are areas between the tooth and gum where bacteria can gather and cause inflammation, can be treated with HA [17]. It may be injected into the pocket to minimize inflammation and stimulate tissue regeneration, resulting in pocket depth reduction and attachment level improvement. HA can be used in periodontal surgery to improve healing and tissue regeneration. It can be either topically or injected into the surgical site to reduce inflammation and promote wound healing. HA is frequently used as a complement to scaling and root planning. It can be used topically or injected into the gum tissue to encourage tissue regeneration and reduce inflammation, hence improving therapy outcomes. It has a high capacity for water binding, which can assist in keeping the gums moist and aid healing. Finally, it has been shown to stimulate fibroblast and other connective tissue cell proliferation, which can help with tissue regeneration. regeneration [18]. Gontiya et al. demonstrated that subgingivally applying 0.2% HA gel with SRP in chronic periodontitis patients improved the gingival index and bleeding index when compared to control sites, which was confirmed by a gingival biopsy, which revealed a significant reduction in inflammatory infiltrate [19]. Eick et al. studied the effect of topical HA association with various molecular weights [20]. In comparison to the control group (SRP only), the HA group showed a positive effect on PPD reduction and the prevention of recolonization by periodontal pathogens such as Campylobacter, P. intermedia, and P. gingivalis. In their clinical trials, Chauhan et al. included 60 patients who were randomly assigned to one of three groups: group 1 got full SRP with subgingival debridement, whereas groups 2 and 3 received topically applied HA gel and chlorhexidine (CHX) gel, respectively, following SRP [21]. During baseline and three-month follow-up, all three groups saw a substantial decrease in PPD and an increase in CAL; however, after three months, the change in PPD and CAL was greater in group 2 than in group 3, although the difference was not statistically significant. Another use for HA in periodontics is as a filler for periodontal problems [22]. To assist in filling gaps and stimulate tissue regeneration, HA can be injected into regions of gum recession or other abnormalities. Because of its anti-inflammatory and regenerating characteristics, HA is a potential biomaterial for use in periodontics. It has the potential to enhance treatment results and minimize the need for more invasive gum disease operations [23].

HA in Pedodontics

It is used in the treatment of oral ulcers; Oral ulcers in children are a frequent condition that can cause discomfort and agony. To assist in decreasing inflammation and improve healing, HA can be used topically or as a mouthwash. In patients with recurrent aphthous ulcers, Nolan et al. proved that using 0.2% HA gel twice daily for two weeks is an effective and safe therapy [24]. Lee et al. investigated the efficacy of 0.2% HA gel topical therapy for oral ulcers in RAU and Behçet's disease patients [25]. It is also used in the management of gingivitis which is a moderate type of gum disease and is frequent in youngsters. To help reduce inflammation and improve gum health, HA can be used as a topical gel or mouthwash. Moreover, in orthodontic therapy, HA can be administered to decrease inflammation and enhance tissue repair. To alleviate discomfort and enhance results, it can be given topically or injected into the soft tissues around the orthodontic device. In the prevention of caries, HA has been researched for its ability to prevent dental caries, a prevalent condition in youngsters. It can be administered topically to the teeth to help reduce bacterial development and promote remineralization [26].

HA in Implant Dentistry

HA has been shown to improve osseointegration and reduce implant failure rates. Studies have reported that the use of HA can result in increased bone density around dental implants, which can improve implant stability and longevity thereby leading to the implant’s long life. Cervino et al. investigated the various surface treatments in titanium implants, demonstrating that biomaterial topography and surface chemistry can correlate with host response [27]. They also focused on the addition of HA to the implant surface and assessed the biological implications during the early stages of recovery. By increasing the connection between implant and bone, HA acts as a coating on the migration, adhesion, proliferation, and differentiation of cell precursors on titanium implants. Additionally, by increasing the bioactivity of the implant surfaces with HA, the dental prosthesis could be accurately positioned during the early loading period, matching the expectations of the patients. The rational use of HA is related to its composition, as it is one of the essential glycosaminoglycans in the cellular matrix synthesized by fibroblasts, synoviocytes, and chondrocytes. It also reduces inflammation during wound healing, promoting cell proliferation, re-epithelialization, and scar reduction.

HA in Endodontics

HA has also been studied for its possible application in endodontics [28]. According to research, using HA after endodontic operations can minimize postoperative discomfort and enhance recovery mainly because of its anti-inflammatory effect [29]. HA has been utilized as an intracanal medicament in endodontics to aid healing and regeneration of the periapical tissues following root canal therapy. One of the primary uses of HA in endodontics is its usage as a root canal irrigant. According to research, HA irrigants may efficiently remove the smear layer and debris from the root canal system while having no negative effects on the dentin or pulp tissue [30]. HA has also been used as a filling material in treating root perforations and resorptive defects. It is a good material for healing these abnormalities due to its biocompatibility and capability to encourage tissue regeneration.

HA in Orthodontics

HA reduces the pain and discomfort associated with orthodontic therapy while simultaneously encouraging faster tooth mobility. Injections of HA into the periodontal ligament have been found to promote osteoclast and osteoblast expression, resulting in greater tooth movement. Gingivitis and periodontitis can occur as a result of orthodontic therapy's increased plaque buildup and inflammation. HA has anti-inflammatory and anti-bacterial properties that may aid in the reduction of inflammation and enhancement of healing in periodontal tissues during orthodontic therapy. To minimize friction between brackets and archwires, HA can be used as a lubricant. This can assist in alleviating pain and make orthodontic therapy more comfortable for patients. HA can be utilized to prevent white spot lesions, which are demineralized regions that can develop around orthodontic brackets. Additionally, the use of HA in orthodontics has the potential to be cost-effective for patients and the whole healthcare system [31].

HA in Facial Aesthetics

A relatively new specialization of maxillofacial surgery is facing aesthetic surgery. Numerous non-surgical techniques are used in facial aesthetics, also known as facial rejuvenation or facial augmentation, to improve the appearance of the face [32]. These treatments can be used to treat ageing symptoms such as wrinkles, fine lines, volume loss, and sagging skin. HA is mainly employed as a dermal filler in facial aesthetics. Due to its capacity to enhance facial contours, restore volume, and generally enhance skin appearance, it has grown in popularity as a cosmetic procedure for facial aesthetics in recent years. HA adds volume and plumps up the targeted area when injected into the skin, which can help fill in wrinkles, folds, and lines [33]. HA dermal fillers are frequently used to add volume to areas like the cheeks, lips, nasolabial folds, and marionette lines (lines that run from the corners of the mouth to the jawline). Injecting HA into the lips can improve their shape and fullness, and it is also employed in other cosmetic aesthetic operations including lip augmentation. To hide bags and dark circles beneath the eyes, it can also be used to fill in the so-called tear troughs. HA fillers can be used to define and contour the jawline, creating a more defined and sculpted appearance. HA is an organic compound that is easily tolerated by the human body, and its effects are typically ephemeral and reversible. This is one benefit associated with using it in facial aesthetics. Depending on the chemical used and the person's metabolism, the health advantages of HA dermal fillers frequently endure for several months to a year [34]. This makes it a popular option for people looking for minor facial augmentations.

Future perspectives on the use of HA in dentistry

Because of the adaptability of HA in modern dentistry, significant potential for the creation of novel and creative dental products and treatments exists. Future research should concentrate on optimizing HA formulations, developing new delivery methods, and investigating new uses for HA in dentistry [35]. To improve its regeneration potential, HA should be used in conjunction with other biomaterials, drugs, and growth factors.

**Table 1:** Summary of the articles included in the review HA: Hyaluronic Acid, TMJ: Temporomandibular Joint, ECM: Extracellular Matrix, SRP: Scaling and Root Planing, CHX: Chlorhexidine

Sr.no.	Authors	Year	Findings
1	Kingsley et al. [1]	2020	Hyaluronic acid fillers can be used as dermal fillers for soft tissue augmentation.
2	Dahiya et al. [2]	2013	Use of HA in periodontal therapies.
3	Valachova et al. [3]	2021	HA in wound healing, tissue engineering, dentistry and gene delivery.
4	Bertl et al. [5]	2015	HA as adjunct to non-surgical and surgical periodontal therapy.
5	Rajan et al. [6]	2014	HA has a beneficial effect on periodontal health in patients with chronic periodontitis.
6	Venilla et al. [7]	2016	Anti-microbial therapy is essential along with conventional therapy in the management of periodontal disease.
7	Lambe et al. [8]	2021	Physiochemical, biochemical, and Pharmaco‑therapeutic uses of HA.
8	Costen [9]	1997	Syndrome of ear and sinus symptoms dependent on TMJ disturbances.
9	Westesson et al. [10]	1985	Disk deformation is closely associated with disturbed joint function.
10	Shapiro [11]	2007	TMJ anatomy
11	Gerry [12]	1954	Effect of trauma and hypermobility on the temporomandibular joint.
12	Casale [13]	2016	Perspectives of hyaluronic acid in dentistry.
13	Rayahin et al. [14]	2015	Influence of high and low molecular weight HA on macrophage activation.
14	Yilmaz et al. [15]	2017	Efficacy of hyaluronic acid in post extraction sockets of impacted molars.
15	Koray et al. [16]	2014	Hyaluronic acid spray on swelling, pain and trismus after surgical extraction of impacted mandibular 3rd molars.
16	Papakonstantinou et al. [17]	2009	The ECM is a three-dimensional fibre mesh, comprised of various interconnected and intercalated macromolecules, among which are the glycosaminoglycans.
17	Bukhari et al. [18]	2018	Hyaluronic acid exhibits remarkable skin regenerating and collagen stimulating efficacy.
18	Gontiya et al. [19]	2012	Subgingival placement of 0.2% HA gel along with SRP provided a significant improvement in gingival parameters.
19	Eick et al. [20]	2013	Hyaluronic acid as an adjunct after scaling and root planning.
20	Chauhan et al. [21]	2013	Comparison of hyaluronan gel and xanthan based CHX gel as an adjunct to scaling and root planning.
21	Pilloni et al. [22]	2011	Evaluation of the efficacy of HA on periodontal clinical parameters.
22	Castrogiovanni et al. [23]	2016	HA as a basic component of ECM and synovial fluid.
23	Nolan et al. [24]	2006	Topical HA efficacy in treatment of recurrent aphthous ulcers.
24	Lee et al. [25]	2008	Effectiveness of topical 0.2% HA gel on ulcers of different nature and cause.
25	Traini et al. [26]	2005	Collagen fiber orientation near dental implants in alveolar bone.
26	Cervino et al. [27]	2021	HA acts on the migration, adhesion, proliferation and differentiation of cell precursors on titanium implants by improving the connection between implant and bone.
27	Akshaya [28]	2018	Regenerative efficacy of 0.2% hyaluronic acid gel in conjunction with chorion membrane in grade ii furcation defect.
28	Bordoni et al. [29]	2022	Head and Neck Anatomy, TMJ.
29	Hashem et al. [30]	2019	Topical hyaluronic acid in the management of oral lichen planus.
30	Nasrollahi et al. [31]	2021	Hyaluronic acid spray for radiation induced mucositis.
31	Freese [32]	1960	TMJ pain disorders.
32	Kochan [33]	1956	Hyaluronidase used in the treatment of temporomandibular joint disturbances.
33	Akbelen et al. [34]	2016	Hyaluronic acid hydrogels for tissue regeneration in oral surgery.
34	Garantziotis et al. [35]	2016	Interfering with the biofilm: antiadhesive and antibiofilm activity of HA.

## Conclusions

HA is a versatile biomaterial with several uses in dentistry. Its function in periodontal treatment, implant dentistry, endodontics, facial aesthetics, oral surgery, and orthodontics has been thoroughly researched. Because of its biocompatibility, regenerative qualities, and antibacterial characteristics, it is an appealing material for use in a variety of dental treatments. Further study is needed to fully understand HA's potential in dentistry and to maximize its usage in clinical practice.

## Appendices

1. Search: Hyaluronic acid[Title/Abstract] AND periodontics[Title/Abstract]

"hyaluronic acid"[Title/Abstract] AND "periodontics"[Title/Abstract]

2. Search: Hyaluronic acid AND oral and maxillofacial surgery

("hyaluronic acid"[MeSH Terms] OR ("hyaluronic"[All Fields] AND "acid"[All Fields]) OR "hyaluronic acid"[All Fields]) AND ("oral maxillofac surg"[Journal] OR "oral maxillofac surg clin north am"[Journal] OR "int j oral maxillofac surg"[Journal] OR ("oral"[All Fields] AND "and"[All Fields] AND "maxillofacial"[All Fields] AND "surgery"[All Fields]) OR "oral and maxillofacial surgery"[All Fields])

3. Search: "Hyaluronic acid" AND "oral medicine"

"Hyaluronic acid"[All Fields] AND "oral medicine"[All Fields]

4. Search: Hyaluronic acid AND orthodontics

("hyaluronic acid"[MeSH Terms] OR ("hyaluronic"[All Fields] AND "acid"[All Fields]) OR "hyaluronic acid"[All Fields]) AND ("orthodontal"[All Fields] OR "orthodontic"[All Fields] OR "orthodontical"[All Fields] OR "orthodontically"[All Fields] OR "orthodontics"[MeSH Terms] OR "orthodontics"[All Fields])

5.  Search: Hyaluronic acid AND endodontics

("hyaluronic acid"[MeSH Terms] OR ("hyaluronic"[All Fields] AND "acid"[All Fields]) OR "hyaluronic acid"[All Fields]) AND ("endodontal"[All Fields] OR "endodontic"[All Fields] OR "endodontical"[All Fields] OR "endodontically"[All Fields] OR "endodontics"[MeSH Terms] OR "endodontics"[All Fields])

6. Search: Hyaluronic acid AND aesthetic dentistry

("hyaluronic acid"[MeSH Terms] OR ("hyaluronic"[All Fields] AND "acid"[All Fields]) OR "hyaluronic acid"[All Fields]) AND (("aesthetical"[All Fields] OR "aesthetically"[All Fields] OR "esthetical"[All Fields] OR "esthetically"[All Fields] OR "esthetics"[MeSH Terms] OR "esthetics"[All Fields] OR "aesthetic"[All Fields] OR "aesthetics"[All Fields] OR "esthetic"[All Fields]) AND ("dentistry"[MeSH Terms] OR "dentistry"[All Fields] OR "dentistry s"[All Fields]))

## References

[1] Kingsley M, Metelitsa AI, Kaminer MS (2013). Injectable dermal and subcutaneous fillers. Comprehensive Dermatologic Drug Therapy.

[2] Dahiya P, Kamal R (2013). Hyaluronic acid: a boon in periodontal therapy. N Am J Med Sci.

[3] Valachová K, Šoltés L (2021). Hyaluronan as a prominent biomolecule with numerous applications in medicine. Int J Mol Sci.

[4] Nutrition SA (2023). Nutrition and Health. https://www.swiss-alp-nutrition.ch/en/.

[5] Bertl K, Bruckmann C, Isberg PE, Klinge B, Gotfredsen K, Stavropoulos A (2015). Hyaluronan in non-surgical and surgical periodontal therapy: a systematic review. J Clin Periodontol.

[6] Rajan P, Baramappa R, Rao NM, Pavaluri AK, P I, Rahaman SM (2014). Hyaluronic Acid as an adjunct to scaling and root planing in chronic periodontitis. A randomized clinical trail. J Clin Diagn Res.

[7] Vennila K, Elanchezhiyan S, Ilavarasu S (2016). Efficacy of 10% whole Azadirachta indica (neem) chip as an adjunct to scaling and root planning in chronic periodontitis: a clinical and microbiological study. Indian J Dent Res.

[8] Lambe S, Ghogare P, Sonawane S, Shinde L, Prashant D (2021). Isolation, purification and characterization of hyaluronic acid: a concise review. J Pharmacogn Phytochem.

[9] Costen JB (1997). A syndrome of ear and sinus symptoms dependent upon disturbed function of the temporomandibular joint. Ann Otol Rhinol Laryngol.

[10] Westesson PL, Bronstein SL, Liedberg J (1985). Internal derangement of the temporomandibular joint: morphologic description with correlation to joint function. Oral Surg Oral Med Oral Pathol.

[11] Shapiro HH (1950). The anatomy of the temporomandibular joint. Oral Surg Oral Med Oral Pathol.

[12] Gerry RG (1954). Effects of trauma and hypermotility on the temporomandibular joint. Oral Surg Oral Med Oral Pathol.

[13] Casale M, Moffa A, Vella P (2016). Hyaluronic acid: perspectives in dentistry. A systematic review. Int J Immunopathol Pharmacol.

[14] Rayahin JE, Buhrman JS, Zhang Y, Koh TJ, Gemeinhart RA (2015). High and low molecular weight hyaluronic acid differentially influence macrophage activation. ACS Biomater Sci Eng.

[15] Yilmaz N, Demirtas N, Kazancioglu HO, Bayer S, Acar AH, Mihmanli A (2017). The efficacy of hyaluronic acid in postextraction sockets of impacted third molars: a pilot study. Niger J Clin Pract.

[16] Koray M, Ofluoglu D, Onal EA, Ozgul M, Ersev H, Yaltirik M, Tanyeri H (2014). Efficacy of hyaluronic acid spray on swelling, pain, and trismus after surgical extraction of impacted mandibular third molars. Int J Oral Maxillofac Surg.

[17] Papakonstantinou E, Karakiulakis G (2009). The 'sweet' and 'bitter' involvement of glycosaminoglycans in lung diseases: pharmacotherapeutic relevance. Br J Pharmacol.

[18] Bukhari SN, Roswandi NL, Waqas M (2018). Hyaluronic acid, a promising skin rejuvenating biomedicine: a review of recent updates and pre-clinical and clinical investigations on cosmetic and nutricosmetic effects. Int J Biol Macromol.

[19] Gontiya G, Galgali SR (2012). Effect of hyaluronan on periodontitis: a clinical and histological study. J Indian Soc Periodontol.

[20] Eick S, Renatus A, Heinicke M, Pfister W, Stratul SI, Jentsch H (2013). Hyaluronic acid as an adjunct after scaling and root planing: a prospective randomized clinical trial. J Periodontol.

[21] Chauhan AS, Bains VK, Gupta V, Singh GP, Patil SS (2013). Comparative analysis of hyaluronan gel and xanthan-based chlorhexidine gel, as adjunct to scaling and root planning with scaling and root planning alone in the treatment of chronic periodontitis: a preliminary study. Contemp Clin Dent.

[22] Pilloni A, Annibali S, Dominici F (2011). Evaluation of the efficacy of an hyaluronic acid-based biogel on periodontal clinical parameters. A randomized - controlled clinical pilot study. Ann Stomatol.

[23] Castrogiovanni P, Trovato FM, Loreto C, Nsir H, Szychlinska MA, Musumeci G (2016). Nutraceutical supplements in the management and prevention of osteoarthritis. Int J Mol Sci.

[24] Nolan A, Baillie C, Badminton J, Rudralingham M, Seymour RA (2006). The efficacy of topical hyaluronic acid in the management of recurrent aphthous ulceration. J Oral Pathol Med.

[25] Lee JH, Jung JY, Bang D (2008). The efficacy of topical 0.2% hyaluronic acid gel on recurrent oral ulcers: comparison between recurrent aphthous ulcers and the oral ulcers of Behçet's disease. J Eur Acad Dermatol Venereol.

[26] Traini T, Degidi M, Strocchi R, Caputi S, Piattelli A (2005). Collagen fiber orientation near dental implants in human bone: do their organization reflect differences in loading?. J Biomed Mater Res B Appl Biomater.

[27] Cervino G, Meto A, Fiorillo L (2021). Surface treatment of the dental implant with hyaluronic acid: an overview of recent data. Int J Environ Res Public Health.

[28] Akshaya N (2018). Evaluation of Regenerative Efficacy of 0.2% Hyaluronic Acid Gel in Conjunction With Chorion Membrane in Grade II Furcation Defect: A Clinical Study. https://www.semanticscholar.org/paper/Evaluation-of-regenerative-efficacy-of-0.2-acid-gel-Akshaya/3bceeab2a333b1163cb4214266a9ec73d43f281f.

[29] Bordoni B, Varacallo M (2023). Anatomy, head and neck, temporomandibular joint. https://pubmed.ncbi.nlm.nih.gov/30860721/.

[30] Hashem AS, Issrani R, Elsayed TE, Prabhu N (2019). Topical hyaluronic acid in the management of oral lichen planus: a comparative study. J Investig Clin Dent.

[31] Nasrollahi H, Khaki S, Ansari M (2021). Evaluation of mucosamin effect on treating radiation induced oral mucositis during and after radiotherapy amongst patients with oral cavity squamous cell carcinoma. Asian Pac J Cancer Prev.

[32] Freese AS (1960). The differential diagnosis of temporomandibular joint pain. Arch Otolaryngol Head Neck Surg.

[33] Kochan EJ (1956). Treatment of temporomandibular joint disturbances with hyaluronidase. Oral Surg Oral Med Oral Pathol.

[34] Kaya ÖA, Muğlali M (2016). The use of hyaluronic acid hydrogels for tissue regeneration in oral surgery: a review. Atatürk Üniv Diş Hekim Fak derg.

[35] Garantziotis S, Brezina M, Castelnuovo P, Drago L (2016). The role of hyaluronan in the pathobiology and treatment of respiratory disease. Am J Physiol Lung Cell Mol Physiol.

